# Regional disparities in breast cancer mortality in Brazil: a spatial analysis using uncorrected and adjusted data, 2000–2023

**DOI:** 10.1038/s41598-026-37844-w

**Published:** 2026-01-30

**Authors:** Juliana Dantas de Araújo Santos Camargo, Sávio Ferreira Camargo, Amaxsell Thiago Barros de Souza, Ana Karla Monteiro Santana de Oliveira Freitas, Camiliane Azevedo Ferreira, Ayane Cristine Alves Sarmento, Janaina Cristiana de Oliveira Crispim, Marcos Roberto Gonzaga, Karina Cardoso Meira, Grasiela Piuvezam, Ana Katherine Gonçalves

**Affiliations:** 1 Postgraduate Program in Health Science, Federal University of Rio Grande do Norte, Natal, RN Brazil; 2 Januário Cicco Maternity School, Brazilian Company of Hospital Services, Natal, RN Brazil; 3 Postgraduate Program in Science Applied to Women’s Health, Federal University of Rio Grande do Norte, Natal, RN Brazil; 4 Institute of Education, Research and Innovation, League Against Cancer, Natal, RN Brazil; 5 Department of Clinical and Toxicological Analysis, Federal University of Rio Grande do Norte, Natal, RN Brazil; 6 Postgraduate Program in Demography, Federal University of Rio Grande do Norte, Natal, RN Brazil; 7 Department of Pharmaceutical Sciences, Federal University of São Paulo, São Paulo, SP Brazil; 8 Department of Public Health, Federal University of Rio Grande Do Norte, Natal, RN Brazil; 9 Systematic Review and Meta-Analysis Laboratory (Lab-Sys/CNPq), Federal University of Rio Grande do Norte, Natal, RN Brazil; 10 Department of Gynecology and Obstetrics, Federal University of Rio Grande do Norte, Natal, RN Brazil

**Keywords:** Health disparities, Breast neoplasms, Mortality, Spatial analysis, Data accuracy, Cancer, Diseases, Health care, Medical research, Oncology

## Abstract

Breast cancer is the leading cause of cancer death among Brazilian women, yet mortality estimates are often underestimated due to ill-defined causes, incomplete diagnoses, underreporting, and data quality limitations. Using national mortality data from 2000 to 2023, we examined the spatial distribution of breast cancer mortality among women aged 20 years and older, comparing uncorrected and adjusted estimates. Adjustments were applied to correct ill-defined causes, incomplete diagnoses, underreporting, and other data quality limitations using methods developed by the World Health Organization and the Brazilian Institute of Geography and Statistics (IBGE). Age-standardized mortality rates were calculated for five time periods using the World Health Organization (WHO) standard population, and spatial patterns were analyzed using choropleth maps, Moran I, and Local Indicators of Spatial Association (LISA). A total of 328,319 breast cancer deaths were reported, increasing to 385,068 (+ 17.3%) after adjustment. Corrections had the greatest effect in the North and Northeast in 2000–2004 (up to + 69.9%), but declined substantially over time. Mortality remained consistently higher in wealthier regions, while adjustments revealed underestimation in historically underserved areas. These findings reveal enduring geographic inequalities in breast cancer mortality and underscore the urgent need for targeted interventions and improved surveillance systems.

**Supplementary Information:**

The online version contains supplementary material available at 10.1038/s41598-026-37844-w.

## Introduction

Breast cancer remains the most commonly diagnosed malignancy and the leading cause of cancer-related mortality among women worldwide, posing significant public health challenges^1^. In Brazil, breast cancer mortality exhibits marked regional disparities, reflecting pronounced socioeconomic inequalities and heterogeneity in healthcare infrastructure and access^2–4^.

While the Southeast and South regions generally report higher breast cancer mortality rates, the lower rates observed in the North and Northeast have often been interpreted as indicating a reduced disease burden. However, growing evidence suggests that these patterns may be influenced by variations in the quality and completeness of mortality data in less developed regions^5,6^.

The Mortality Information System (SIM) is the primary source of mortality data in Brazil. Although it has undergone substantial improvements in recent decades, important limitations persist, including deaths classified under ill-defined causes, incomplete diagnostic information, and other data quality issues (such as underreporting, population under-enumeration, and age misreporting) that disproportionately affect less developed areas^7,8^. These limitations can lead to underestimation of cause-specific mortality rates, compromise the accuracy of temporal trend analyses, and obscure true regional inequalities^9–11^.

Spatial epidemiology provides powerful tools for visualizing and analyzing the geographic distribution of diseases, identifying clusters, and investigating associations with contextual determinants^12,13^. Although several studies have examined spatial patterns of breast cancer mortality in Brazil, few have incorporated adjustments for data quality issues, which may result in underestimation of the disease burden in specific regions^14,15^.

In this context, the present study aims to describe the spatial distribution of breast cancer mortality across Brazilian states using both uncorrected data and data adjusted for ill-defined causes, incomplete diagnoses, and other data quality issues, over the period from 2000 to 2023.

### Results

#### Overall breast cancer mortality burden and impact of data adjustments

**Fig. 1:**
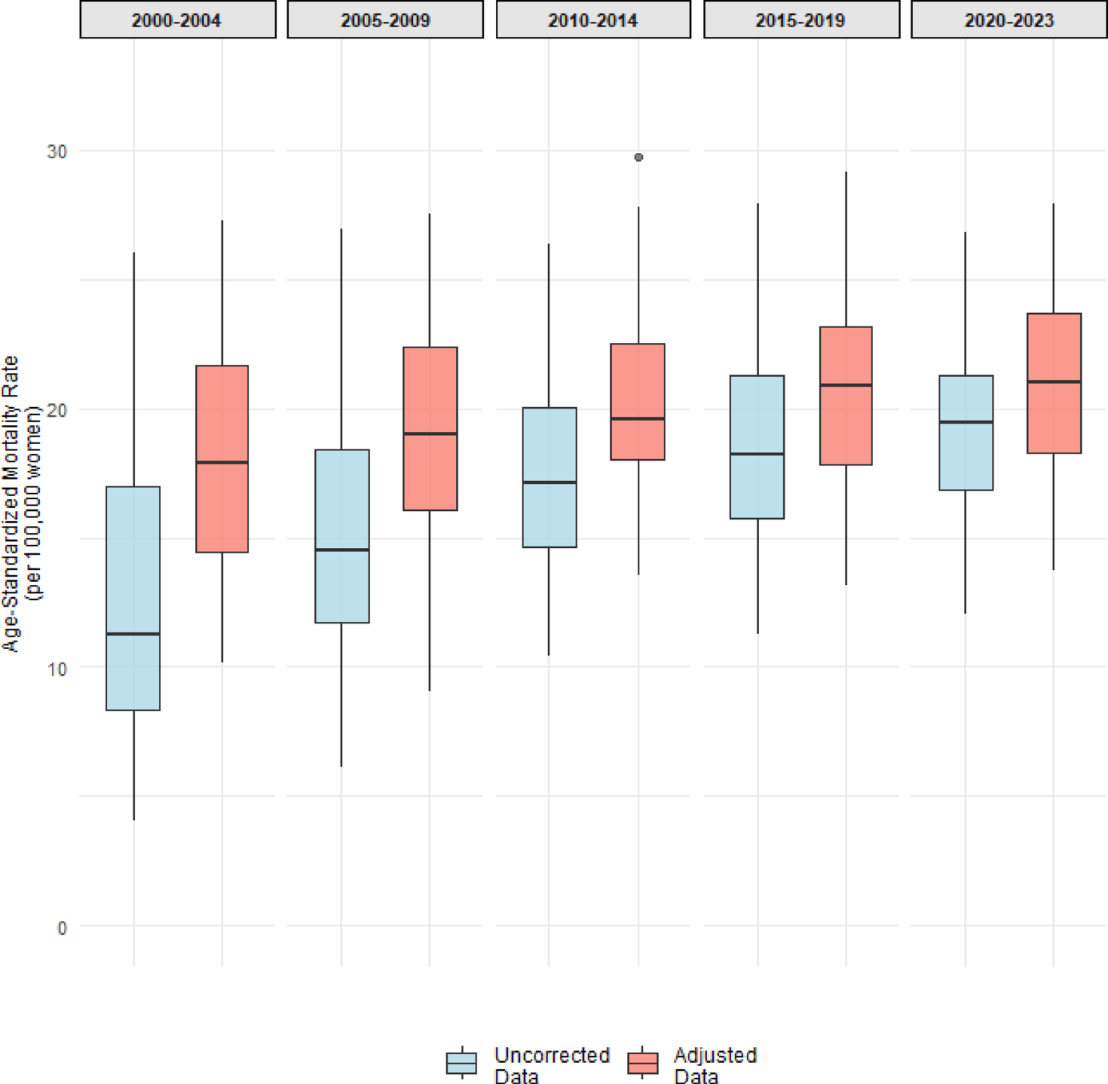
Distribution of age-standardized breast cancer mortality rates across Brazilian states: uncorrected vs. adjusted data by period, 2000–2023.

Between 2000 and 2023, Brazil officially recorded 328,319 deaths attributed to breast cancer. After adjusting for ill-defined causes, incomplete diagnoses, and data quality issues, the estimated number of deaths rose to 385,068, representing a 17.3% increase compared to the originally reported figures. Figure 1 illustrates the distribution of age-standardized breast cancer mortality rates across Brazilian states for each time period (2000–2023), comparing uncorrected and adjusted data.

Median uncorrected rates ranged from 11.24 (IQR = 8.61) to 19.46 (IQR = 4.48) per 100,000 women, while median adjusted rates ranged from 17.92 (IQR = 7.19) to 20.99 (IQR = 5.39) per 100,000 women. Both uncorrected and adjusted rates showed an upward trend over time, with adjusted rates consistently exceeding uncorrected rates, particularly in earlier periods (2000–2009) (Fig. 1).

Table 1 presents the average age-standardized breast cancer mortality rates for Brazil and its macro-regions across five time periods from 2000 to 2023, based on both uncorrected and adjusted data. Adjusted estimates account for ill-defined causes, incomplete diagnostic coding, and data quality issues.

**Table 1:** Average age-standardized breast cancer mortality rates in Brazilian regions: uncorrected and adjusted data, 2000–2023.^1^ Data are expressed as mean ± standard deviation.

Brazilian region	Period	Uncorrected	Adjusted	Relative change (%)
North	2000–2004	8.25 ± 1.15	14.02 ± 2.28	69.92
Northeast		9.97 ± 3.36	16.73 ± 3.81	67.77
Midwest		16.08 ± 4.86	19.44 ± 5.24	20.85
Southeast		20.12 ± 5.81	22.75 ± 5.02	13.06
South		20.85 ± 3.77	25.20 ± 1.48	20.85
Brazil		**13.14 ± 6.09**	**18.26 ± 5.14**	**38.94**
North	2005–2009	9.73 ± 2.58	13.78 ± 3.25	41.67
Northeast		13.98 ± 3.16	18.64 ± 3.25	33.27
Midwest		17.73 ± 3.83	20.85 ± 3.93	17.62
Southeast		20.53 ± 5.24	23.02 ± 4.87	12.09
South		20.73 ± 3.20	24.15 ± 0.97	16.50
Brazil		**15.16 ± 5.26**	**18.97 ± 4.87**	**25.15**
North	2010–2014	12.63 ± 1.88	15.71 ± 2.35	24.41
Northeast		16.04 ± 2.80	19.41 ± 2.83	21.02
Midwest		19.83 ± 3.23	22.43 ± 4.96	13.12
Southeast		21.20 ± 4.01	23.34 ± 3.15	10.14
South		22.10 ± 1.96	24.72 ± 2.71	11.84
Brazil		**17.15 ± 4.36**	**20.07 ± 4.31**	**17.01**
North	2015–2019	15.08 ± 1.44	16.95 ± 1.36	12.43
Northeast		17.61 ± 3.29	19.94 ± 3.42	13.22
Midwest		20.71 ± 1.83	23.59 ± 4.62	13.91
Southeast		22.29 ± 4.15	24.04 ± 3.23	7.83
South		22.81 ± 1.87	24.35 ± 2.88	6.75
Brazil		**18.69 ± 3.86**	**20.81 ± 4.10**	**11.34**
North	2020–2023	15.97 ± 3.26	17.75 ± 3.46	11.12
Northeast		18.18 ± 2.86	20.37 ± 2.89	12.06
Midwest		20.16 ± 0.92	22.41 ± 4.40	11.18
Southeast		21.94 ± 3.68	23.85 ± 3.35	8.70
South		22.87 ± 1.46	24.78 ± 3.12	8.35
Brazil		**18.98 ± 3.57**	**21.00 ± 3.98**	**10.64**

Significant values are in bold

#### Regional patterns of mortality adjustments

In all periods, average mortality rates were higher in the adjusted dataset compared to the uncorrected one across all regions. The magnitude of the adjustments varied by region and period. The largest relative increase was observed in the North Region during 2000–2004, with adjusted rates 69.9% higher than uncorrected values. Similarly, in the Northeast, the adjustment in the same period reached 67.8%. In contrast, the Southeast and South regions showed smaller percentage changes, particularly in the later periods. Nationally, the adjusted mortality rate rose from 13.14 (± 6.09) to 18.26 (± 5.14) per 100.000 women in 2000–2004, representing a 38.9% increase. By the most recent period (2020–2023), this difference had declined to 10.6%, with rates increasing from 18.98 (± 3.57) to 21.00 (± 3.98) per 100.000 women (Table 1).

#### State-level differences and magnitude of corrections

Table S1 presents age-standardized breast cancer mortality rates for all Brazilian states across five time periods from 2000 to 2023, including both uncorrected and adjusted estimates. Adjusted rates were consistently higher than their uncorrected counterparts across all states and periods. The magnitude of these differences varied substantially by location and time, with the most pronounced corrections observed in the early 2000s. Statistically significant differences between uncorrected and adjusted values were observed for every study period (Wilcoxon signed-rank test, *p* < 0.001 for all five periods).

Between 2000 and 2004, states such as Maranhão, Piauí, Amazonas, and Pará exhibited adjusted mortality rates that were more than 80% higher than the uncorrected estimates. In Maranhão, for example, the rate increased from 4.01 to 10.14 deaths per 100,000 women. Similarly, the rate rose from 8.39 to 15.63 in Pará and from 9.26 to 16.79 in Amazonas. In contrast, more developed states—including São Paulo, Rio de Janeiro, and the Federal District—consistently showed much smaller discrepancies throughout the entire period. For example, in São Paulo, the adjusted rate for 2020–2023 was 27.46, compared to an uncorrected rate of 22.53. Over time, the magnitude of the correction decreased in most Brazilian states, and the difference between adjusted and uncorrected rates remained below 20% in the most recent period. (Table S1).

#### Spatial patterns of breast cancer mortality

**Fig. 2:**
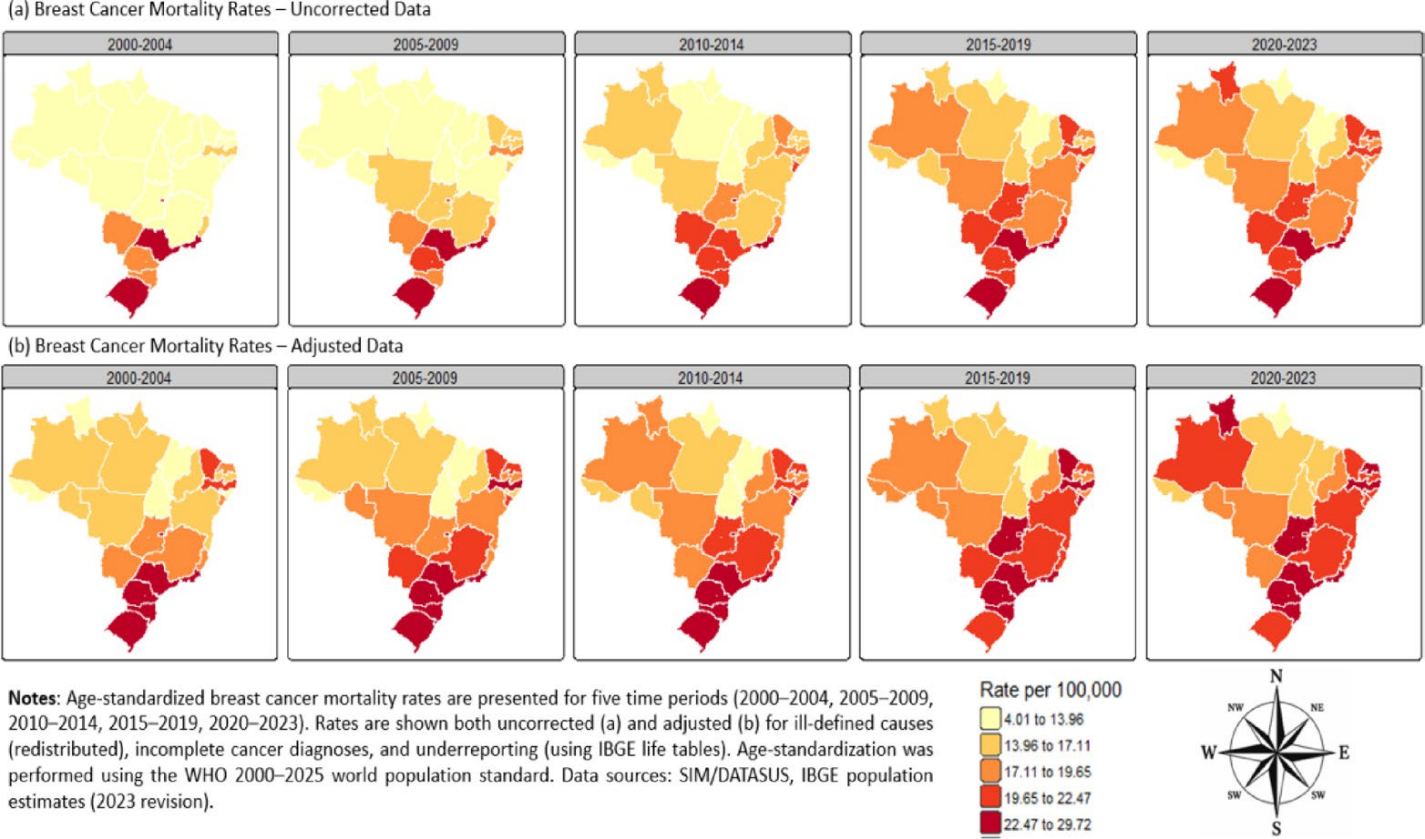
Spatial patterns of breast cancer mortality rates in Brazilian States: uncorrected vs. adjusted data by period, 2000–2023.

Figure 2 presents choropleth maps of breast cancer mortality by state and period, comparing uncorrected and adjusted data. Spatial heterogeneity in mortality patterns was evident throughout the study period, with persistently high mortality rates concentrated in the Southeast and South regions, while lower rates prevailed in the North and Northeast. The adjusted maps revealed previously underestimated mortality burdens in several states in the North, particularly during the earlier periods (2000–2009).

Visual inspection of the maps suggests elevated breast cancer mortality in more developed regions, such as the Southeast and South. However, the adjusted maps also highlight emerging hotspots in specific states in the North and Northeast regions over time (Fig. 2).

#### Spatial autocorrelation analysis

Global spatial autocorrelation of age-standardized breast cancer mortality rates, assessed using Moran I test, did not reveal any statistically significant (*p* > 0,05) spatial clustering across the five study periods, regardless of data correction status. Moran I values ranged from − 0.739 to 0.251, with all corresponding *p*-values exceeding 0.40, indicating a random spatial distribution of mortality rates among Brazilian states from 2000 to 2023. These values were consistent for both uncorrected and adjusted rates, indicating no evidence of large-scale spatial patterns in breast cancer mortality—even after adjusting for ill-defined causes, incomplete diagnostic codes, and data quality issues (Table 2).

**Table 2:** Global Spatial Autocorrelation of Age-Standardized Breast Cancer Mortality Rates in Brazilian States, by Period and Correction Status, 2000–2023.

Period	Uncorrected		Adjusted	
	Moran I	p-value	Moran I	*p*-value
2000–2004	− 0.489	0.687	− 0.353	0.704
2005–2009	− 0.025	0.509	0.087	0.465
2010–2014	− 0.179	0.571	− 0.169	0.567
2015–2019	− 0.430	0.667	− 0.739	0.770
2020–2023	0.251	0.401	− 0.502	0.692

Global Moran I statistic was used to assess spatial clustering of age-standardized breast cancer mortality rates across Brazilian states. No statistically significant clustering was observed in any period, regardless of data correction for ill-defined causes, incomplete diagnoses, or data quality issues (all *p* > 0.40).

#### Local indicators of spatial association (LISA)

**Fig. 3:**
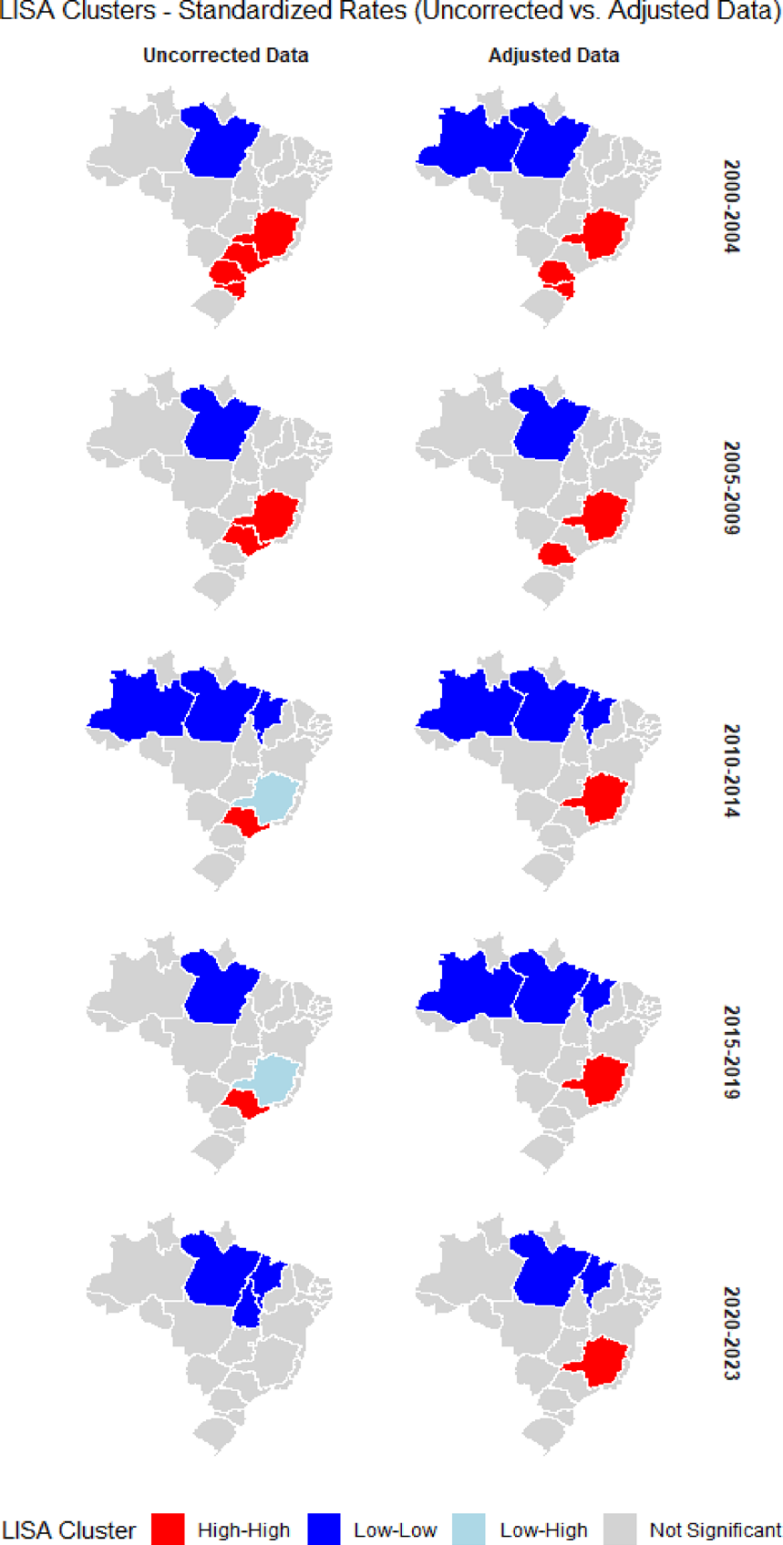
Spatial clustering of breast cancer mortality in Brazil: LISA results for uncorrected and adjusted rates, 2000–2023.

Despite the absence of statistically significant global spatial autocorrelation, local spatial analysis (LISA) revealed distinct and consistent clustering patterns across study periods and between uncorrected and adjusted datasets (Fig. 3). High–high clusters (hotspots), areas with high mortality rates surrounded by neighbors with similarly high rates—were identified in the Southeast and South regions, particularly in the states of Minas Gerais, São Paulo, and Paraná. These hotspots persisted across all time intervals in the adjusted series for Minas Gerais, highlighting a consistently high burden of breast cancer mortality in this state.

Conversely, low–low clusters (coldspots), areas with low mortality rates surrounded by similarly low-rate neighbors, were predominantly observed in the North and Northeast regions, including the states of Amazonas, Pará, and Maranhão. Low–high patterns, indicative of spatial outliers, emerged only in the state of Minas Gerais for uncorrected data during the 2010–2019 period. Importantly, data correction altered the cluster classifications in some states that were previously non-significant (Fig. 3).

### Discussion

Our analysis revealed that, between 2000 and 2023, the spatial distribution of breast cancer mortality in Brazil consistently showed higher rates in the South and Southeast regions, whereas the North and Northeast generally exhibited lower values. However, relative to the original unadjusted mortality estimates, the adjusted data indicated that breast cancer mortality had been previously underestimated in several states of the North and Northeast, particularly in the early years of the study period. Corrections for ill-defined causes, incomplete cancer diagnoses, and data quality issues not only increased overall mortality estimates but also modified spatial patterns, highlighting previously obscured high-risk areas. Although no statistically significant global spatial autocorrelation was detected, local analysis consistently identified clusters of high mortality in parts of the South and Southeast and clusters of low mortality in areas of the North and Northeast, with some classifications changing after data correction.

Our findings are consistent with previous studies documenting substantial regional disparities in breast cancer mortality in Brazil, largely reflecting differences in socioeconomic status, access to healthcare services, and disparities in screening coverage, diagnostic access, and treatment availability^2,13,16^. Persistently higher mortality rates in the Southeast and South likely reflect improvements in cancer registry systems, combined with greater exposure to risk factors associated with urbanization and population aging. In contrast, historically lower rates in the North and Northeast have often been interpreted as indicative of a lower disease burden, but are increasingly recognized as underestimations resulting from incomplete data and weaknesses in healthcare infrastructure^5,6,9^.

The observed decline in the magnitude of corrections over time suggests improvements in death certification and coverage of the SIM, particularly following investments in strengthening vital statistics in Brazil during the 2000s and 2010s^17^. Nonetheless, even in recent years, differences exceeding 10% between unadjusted and adjusted mortality rates persisted in most states, indicating that data quality remains a challenge. The adjusted mortality estimates presented in our study demonstrate that underreporting and ill-defined cause-of-death codes disproportionately affect less developed regions, masking the true magnitude of the burden and reinforcing inequalities in resource allocation and policy targeting^11,18^. These findings underscore the need to strengthen health information systems, enhance cancer screening programs, and improve access to timely, high-quality treatment—particularly in socioeconomically vulnerable areas such as the North and Northeast regions—to reduce mortality disparities and achieve equitable outcomes in cancer control^14,19^.

Part of the changes observed in the LISA patterns (Fig. 3), both across periods and in the comparison between uncorrected and corrected data, reflects genuine temporal dynamics, but another part is clearly influenced by improvements in data quality and by the correction procedures applied to the series. The Low–Low cluster (blue), predominantly located in the North and Northeast regions, shows a noticeable expansion in the corrected data, particularly between 2000–2004/2010–2014 and 2015–2019, whereas a reduction is observed in 2005–2009 and 2020–2023. This fluctuation indicates that these states consistently exhibit breast cancer mortality rates below the national average but lie near the threshold for statistical significance; thus, small variations in the rates after correction may be sufficient to include or exclude them from the Low–Low cluster. This temporal fluctuation is therefore less indicative of abrupt epidemiological changes and more a reflection of improvements in death reporting coverage and classification quality over two decades, which gradually reveal mortality rates that are closer to reality^7,8^ [REF].

A similar mechanism explains the alternation observed between high–high clusters (red) and non-significant areas (gray) in 2005–2009 when comparing unadjusted and adjusted data. In this period, the correction procedures increase mortality rates in some neighboring states and reduce local contrasts, causing certain states to no longer form a statistically significant high-risk cluster, while others are classified as high–high only after adjustment. In the uncorrected data, the apparent expansion of low–low clusters in the North region between 2005–2009, 2010–2014, and 2015–2019 could suggest a widening of regional inequalities; however, the adjusted maps reveal a more stable pattern, with attenuation of this artificial “protective” effect. At the same time, high–high clusters remain concentrated in a few states in the South/Southeast, reinforcing that part of the spatial gradient observed in crude rates is spurious and stems from limitations in the quality and coverage of mortality information systems. These findings underscore the importance of applying correction procedures when comparing historical series in contexts marked by inequalities in data quality^20–22^.

Furthermore, the identification of localized clusters of high breast cancer mortality in the Southeast and South regions, even after data correction, suggests the presence of persistent high-risk areas that may be influenced by regional variations in environmental exposures, lifestyle factors, healthcare access and, possibly, genetic susceptibility^15,23,24^. Although genetic predisposition has been hypothesized as a contributing factor, region-specific genomic studies remain limited, and this explanation should therefore be interpreted as a hypothesis requiring further investigation^25,26^. Conversely, clusters of lower mortality in the North and Northeast that shifted substantially after adjustment underscore how data quality limitations, particularly high levels of ill-defined causes, incomplete cancer coding, and data quality issues, can artificially suppress mortality rates and obscure the true spatial distribution of risk^7,12^. This pattern also helps explain why, despite adjustments, wealthier regions continue to show higher mortality: it is likely that part of the apparent regional contrast reflects underestimation in underserved areas rather than genuinely lower risk.

Overall, these findings are consistent with spatiotemporal analyses from other countries showing that disparities in cancer mortality are shaped by multifactorial determinants operating across different geographic scales^27,28^. Therefore, targeted interventions should be context-specific, addressing social determinants of health in underserved areas while focusing on lifestyle factors, early detection, and quality of care in wealthier regions with higher observed mortality. This approach ensures that strategies are aligned with the actual local risk patterns, reflecting both socioeconomic disparities and region-specific risk factors.

It is important to emphasize that our study reinforces the value of improving the quality of mortality data through systematic correction methods, which enable the identification of underreporting and biases in death records, enhance epidemiological understanding, and support more effective public health planning^7,9,29^. While such adjustments increase confidence in mortality estimates, challenges remain, including heterogeneity in the quality of death certification across Brazilian states and over time^30,31^. Moreover, improvements in data quality alone may not fully offset the structural inequalities in healthcare access and socioeconomic conditions that drive disparities in breast cancer mortality^18,32^. Future research should integrate spatial epidemiological analyses with sociodemographic information and data on healthcare utilization to deepen the understanding of factors influencing breast cancer outcomes and to inform policies aimed at promoting equity in prevention and treatment.

Beyond data correction and the identification of spatial disparities, our findings have important implications for public health policies aimed at reducing breast cancer mortality in Brazil. The persistence of regional inequalities underscores the urgency of ensuring equitable resource allocation, including expanding mammography coverage, strengthening primary care for early detection, and improving referral systems to specialized oncology centers^16,19^. Social determinants such as education, income, and urbanization should be integrated into cancer control strategies, recognizing that populations in the North and Northeast regions face heightened vulnerabilities that hinder timely diagnosis and treatment adherence^4,33,34^. Furthermore, investment in health information systems to standardize and improve the quality of death certification is essential for enabling reliable monitoring and evaluation of interventions^11,32^. Addressing these challenges at multiple levels requires coordinated efforts among federal, state, and municipal health authorities, alongside community engagement, to raise awareness of breast cancer and reduce barriers to treatment.

Our spatial analysis underscores that breast cancer mortality in Brazil cannot be fully understood through aggregated national statistics alone; it requires regionally and locally nuanced perspectives that account for the interplay of epidemiological, social, and healthcare system factors. By revealing underdiagnosed mortality burdens and altering risk patterns through data correction, this study contributes to a more accurate and equitable epidemiological picture. Sustained surveillance with high-quality data, coupled with targeted public health initiatives, will be essential to reducing disparities in breast cancer mortality and improving outcomes for all Brazilian women.

This study has some limitations. The ecological design limits causal inference at the individual level and is subject to the modifiable areal unit problem (MAUP), which may influence spatial clustering results^35^. Regional heterogeneity in registry completeness and the quality of death certification may also affect the magnitude and spatial distribution of breast cancer mortality. In areas with higher levels of ill-defined causes, incomplete cancer coding, or data quality issues, mortality may remain underestimated even after correction, influencing the persistence or disappearance of mortality clusters. Although advanced correction procedures were applied, potential bias from the redistribution of incomplete cancer diagnoses should be acknowledged. While this is a widely used approach, it assumes that incomplete cancer diagnoses follow the same proportional distribution as fully coded neoplasm deaths, which may not hold uniformly across regions with different diagnostic capacity or coding practices. Consequently, residual misclassification of causes of death may persist, particularly in regions with poorer data quality^7,9^.

Furthermore, temporal changes in the availability and quality of healthcare services over the study period may also have influenced the observed trends and spatial patterns^36^. Future ecological studies adopting finer spatial scales—such as municipalities or health regions—and incorporating temporal analyses could provide a more detailed understanding of breast cancer mortality, enabling the exploration of intra-regional variations and the identification of smaller high-risk clusters.

Despite these limitations, this study has several notable strengths. By applying comprehensive correction methods to a large, nationally representative mortality database spanning two decades, we address broader data quality issues or registry limitations that persist across multiple periods, including underreporting of deaths, population under-enumeration, and age misreporting. Correcting for these issues provides more accurate and reliable estimates of breast cancer mortality. In addition, the use of local spatial statistics enabled the identification of clusters and changes in risk that aggregated analyses might overlook, thereby informing targeted public health interventions. Our findings help to fill important knowledge gaps regarding the true burden of breast cancer mortality in socioeconomically vulnerable regions, supporting efforts to improve equity in cancer control across Brazil.

### Conclusions

Our study highlights significant regional disparities in breast cancer mortality across Brazil, with adjusted data revealing previously underestimated burdens in socioeconomically vulnerable areas. The spatial patterns identified provide a clearer understanding of mortality distribution and the impact of data quality on epidemiological estimates. These findings contribute to a more accurate and comprehensive assessment of breast cancer burden, offering important insights for future research and evidence-based public health planning.

## Methods

### Study population and data source

This ecological study employed a spatial approach to analyze breast cancer mortality rates among women aged 20 years or older, residing in Brazil, who died between 2000 and 2023 with breast cancer listed as the underlying cause of death. The inclusion of women from the age of 20 was based on the low incidence of the disease before this age group^37^. Data on female breast cancer deaths were obtained from the Mortality Information System (SIM/DATASUS)^38^. Records of interest were classified under code C50, referring to malignant neoplasms of the breast, according to the 10th Revision of the International Classification of Diseases (ICD-10). Annual population estimates by age group and state (Federative Unit, UF) were retrieved from the Brazilian Institute of Geography and Statistics (IBGE), based on intercensal projections^39^.

### Calculation of mortality rates

Age-specific breast cancer mortality rates (ICD-10: C50) were calculated for each UF over five periods: four five-year intervals (2000–2004, 2005–2009, 2010–2014, 2015–2019) and one four-year interval (2020–2023). The numerator consisted of the mean annual number of breast cancer deaths in each period to reduce random annual fluctuations. The denominator population was based on the estimated population at the midpoint of each period, according to the IBGE 2024 intercensal revision. For the five-year periods, the central year population was used (e.g., 2002 for 2000–2004). For the final four-year period (2020–2023), the population was interpolated to January 1, 2022, using the mean of the estimated populations for 2021 and 2022.

Rates were age-standardized using the direct method, with the World Health Organization (WHO) standard population for 2000–2025 as the reference^40^ and expressed per 100.000 women.

### Correction of death records

Despite substantial improvements in the Mortality Information System (SIM) over recent decades, a considerable proportion of death records, particularly in the North and Northeast regions, continues to be affected by data quality issues that undermine the reliability of long-term spatial analyses. These limitations arise from three major sources: ill-defined causes of death (garbage codes), incomplete diagnostic coding for cancer deaths, and broader registry limitations related to mortality and population data^7,9,41–43^.

First, ill-defined causes of death (“garbage codes”) include deaths for which the underlying cause is classified as vague, nonspecific, or implausible as a true underlying cause. These correspond primarily to ICD-10 Chapter XVIII (R00–R99) and other codes representing symptoms, signs, or terminal events that should not be considered underlying causes. High levels of garbage codes reflect poor medical certification quality and lead to systematic underestimation of specific causes such as breast cancer mortality^8,44^.

Second, incomplete diagnostic coding for cancer deaths (missing or unspecified neoplasm codes) refers to deaths assigned to ICD-10 C76–C80 and C97, which indicate a malignant neoplasm of unspecified site or disseminated cancer without identifying the primary tumor. Because these deaths represent true cancer cases with incomplete site specification, proportional redistribution is required to avoid underestimating site-specific mortality, including breast cancer^45^.

Third, broader data quality issues or registry limitations persist across multiple periods, including underreporting of deaths, population under-enumeration, and age-misreporting. Unlike garbage codes or unspecified cancer diagnoses, these factors affect all cause-specific mortality rates because they distort both the numerator (death counts) and denominator (population at risk). These problems disproportionately affect less-developed regions and necessitate adjustment of overall mortality totals and population estimates^43,46,47^.

These adjustment factors were selected based on three criteria: (i) they correspond to the core components of the WHO and GBD correction frameworks for cause-of-death estimation^48,49^ and the Global Burden of Disease (GBD) Study^50–52^; (ii) Brazilian literature demonstrates that these issues are the predominant sources of bias in cancer mortality statistics^9,22,53^; and (iii) they have direct relevance for breast cancer, for which misclassification into garbage codes or unspecified neoplasm categories is well documented^10,54^.

Additional potential sources of misclassification were not evaluated, including sex misclassification, age–cause incompatibility, underreporting of home deaths, and incorrect selection of the underlying cause due to poorly completed death certificates^52,55,56^. These limitations typically require medical record review or linkage with complementary health information systems, which are not available nationwide and therefore could not be systematically assessed.

To address the main limitations associated with the adjustment used in this study (ill-defined causes, incomplete cancer diagnoses and data quality issues), we applied a correction methodology that combined the WHO approach^57,58^ with demographic adjustments developed by the IBGE^39^. This correction process involved four steps:

1. Redistribution of ill-defined causes: Half of the deaths classified as ill-defined causes were proportionally redistributed among known natural causes, excluding external causes, by year and age group.
2. Redistribution of incomplete cancer diagnoses: Deaths with incomplete cancer diagnoses (codes C76–C80 and C97) were redistributed proportionally across neoplasm categories (ICD-10 Chapter II) by year and age group^22^.
3. Correction of mortality data quality issues: To correct the inconsistencies in mortality records, we used the female life tables from the IBGE 2024 population projections, which incorporate adjustments for underreporting of deaths, population under-enumeration, and age misreporting through established demographic methods^39^. First, age-specific female all-cause mortality rates (nMx) were calculated by UF, year, and age group from SIM data. Then, corresponding nMx values from IBGE life tables were obtained. The correction factor (CF) was calculated as the ratio of the IBGE nMx to the observed nMx (CF = nMx_IBGE / nMx_SIM). This CF was applied to SIM-recorded breast cancer deaths, already adjusted for ill-defined causes and incomplete diagnoses, to proportionally align the counts with IBGE’s demographic corrections.
4. Integration of correction steps: Results from the three previous steps — redistribution of ill-defined causes, redistribution of incomplete diagnoses, and correction of mortality inconsistencies — were integrated into the original number of breast cancer deaths recorded in SIM/DATASUS.

### IBGE life tables and nMx correction factor

The IBGE life tables, particularly those from the 2024 revision of population projections, provide adjusted estimates of age-specific mortality rates (nMx) for women, by UF, year, and age group. These tables were produced using the demographic components method, which integrates mortality, fertility, and migration data, along with statistical and demographic models to correct for the effect of underreporting of deaths, under-enumeration of population and age misreporting on both deaths records and population census^39,43^.

To correct for those inconsistencies in the age-specific mortality risk due to breast cancer deaths in SIM, the following approach was applied:

1. Calculation of observed rates: Age-specific female mortality rates (nMx) were calculated for each UF, year, and age group using SIM data.
2. Extraction of reference rates: Corresponding nMx values were obtained from IBGE life tables, considered the most reliable and corrected reference for general female mortality.
3. Calculation of the correction factor (CF): For each UF, year, and age group, the CF was computed as the ratio between the IBGE life table nMx and the observed SIM nMx:
  $$CF=\frac{nM{x}_{IBGE}}{nM{x}_{SIM}}$$
4. Application of the correction factor: This CF was applied to the number of breast cancer deaths in SIM after they had been redistributed for ill-defined causes and incomplete cancer diagnoses, proportionally adjusting the counts according to the IBGE official demographic corrections. It is important to emphasize that the CF represents a correction factor aimed at addressing those inconsistencies in death records and population censuses, rather than solely reflecting the underreporting of deaths.

### Statistical analysis

Analyses compared uncorrected and adjusted age-standardized breast cancer mortality rates, with adjusted estimates accounting for ill-defined causes, incomplete diagnoses, and data quality issues. All procedures were conducted for each of the five study periods. Descriptive statistics comprised means, standard deviations, medians, interquartile ranges (IQR), and relative frequencies. The relative difference between adjusted and uncorrected rates was calculated as:

$$\text{Relative Difference }(\mathrm{\%})=\left(\frac{{{Rates}_{Adjusted}-Rates}_{Uncorrected}}{{Rates}_{Uncorrected}}\right)x100$$

Differences between adjusted and uncorrected rates were tested using the Wilcoxon signed-rank test. Box plots were used to depict rate distributions across periods. For each period, choropleth maps of mean adjusted and uncorrected rates were produced to illustrate their spatial distribution across Brazilian states (UFs), based on official IBGE shapefiles and generated with the ggplot2, sf, and tmap packages in R (version 4.5.1).

### Spatial analysis

We evaluated spatial autocorrelation using formal global and local spatial statistics, incorporating a mathematical formulation for Moran *I* and Local Moran *I* (LISA)^59–61^. Both the Global Moran *I* and LISA were computed using a spatial proximity (neighborhood) matrix *W*. For a set of *n* regions (*R*_*1*_, …, *R*_*n*_), each element w_ij_ represents a measure of spatial proximity between regions *R*_*i*_ and *R*_*j*_. A first-order contiguity spatial weights matrix was constructed using the Queen criterion to define neighboring states.

### Global spatial autocorrelation: Moran I

The Global Moran *I* measures the degree of spatial autocorrelation of the variable of interest across all regions in the study area, providing a single summary statistic for the entire spatial domain. The index is calculated using the following formula:

$$I= \frac{{\sum }_{i=1}^{n}\sum_{j=1}^{n}{w}_{ij}({z}_{i}-\overline{z })({z}_{j}-\overline{z })}{\sum_{i=1}^{n}{({z}_{i}-\overline{z })}^{2}}$$

where *z*_*i*_ is the mortality rate in region i, *z* is the overall mean, and *w*_*ij*_ represents spatial proximity. Values of Moran *I* range from − 1 to + 1:

- *I* ≈ 0 → spatial randomness
- *I* > 0 → positive spatial autocorrelation (clustered patterns)
- *I* < 0 → negative autocorrelation (spatial dispersion)

This measure evaluates whether the overall spatial pattern reflects clustering or randomness across Brazil.

### Local spatial autocorrelation: local Moran I (LISA)

The Local Moran *I* assesses the spatial autocorrelation between each region and its neighboring regions and, unlike the Global Moran *I*, is expressed as a separate value for every region in the study area. For region (i):

$${I}_{i}= \frac{{z}_{i}{\sum }_{j=1}^{n}{w}_{ij}{z}_{j}}{\sum_{j=1}^{n}{z}_{j}^{2}}$$

From ${I}_{i}$, we constructed LISA cluster maps identifying five conventional categories:

- High–High (HH): high-rate region surrounded by high-rate neighbors
- Low–Low (LL): low-rate region surrounded by low-rate neighbors
- High–Low (HL): high-rate region surrounded by low-rate neighbors (spatial outlier)
- Low–High (LH): low-rate region surrounded by high-rate neighbors (spatial outlier)
- Not significant (NS): no statistically significant local autocorrelation

Statistical significance of LISA clusters was determined using 999 Monte Carlo permutations, with *p* < 0.05 considered significant. All spatial analyses were performed in R software (version 4.5.1) using the packages spdep, sf, spatialreg, and ggplot2.

### Ethics

Only aggregated, public, secondary data without individual identification were used, exempting this study from submission to a Research Ethics Committee, in accordance with Brazilian National Health Council Resolution No. 510/2016.

## Supplementary Information

Below is the link to the electronic supplementary material.

Supplementary Material 1

## Data Availability

The datasets generated during and/or analysed during the current study are available from the corresponding author on reasonable request.

## Abbreviations

IBGE: Brazilian Institute of Geography and Statistics
SIM: Mortality information system
DATASUS: Department of Informatics of the Brazilian Unified Health System
ICD-10: 10Th revision of the international classification of diseases
UF: Federative unit
WHO: World Health Organization
nMx: Age-specific mortality rate for the age interval n
CF: Correction factor
IQR: Interquartile ranges
LISA: Local indicators of spatial association
MAUP: Modifiable areal unit problem
