# Supplementary material for: Regional disparities in breast cancer mortality in Brazil: a spatial analysis using uncorrected and adjusted data, 2000–2023
Source: Sci Rep. 2026 Jan 30;16:6770. doi: 10.1038/s41598-026-37844-w (PMC12913644; doi:10.1038/s41598-026-37844-w)
Supplement: Supplementary file 1 — Supplementary Material 1 [file 41598_2026_37844_MOESM1_ESM.docx]

| Region | UF | 2000–2004 | | 2005–2009 | | 2010–2014 | | 2015–2019 | | 2020–2023 | |
| --- | --- | --- | --- | --- | --- | --- | --- | --- | --- | --- | --- |
|  |  | Uncorrected | Adjusted | Uncorrected | Adjusted | Uncorrected | Adjusted | Uncorrected | Adjusted | Uncorrected | Adjusted |
| North | RO | 9.9 | 15.68 | 13.04 | 17.58 | 11.3 | 14.02 | 15.73 | 17.82 | 15.93 | 17.86 |
|  | AC | 6.47 | 11.03 | 6.13 | 9.76 | 13.58 | 16.36 | 15.6 | 17.52 | 13.36 | 15.09 |
|  | AM | 9.26 | 16.79 | 10.7 | 15.63 | 11.65 | 13.88 | 13.27 | 14.59 | 19.17 | 20.99 |
|  | RR | 7.24 | 11.02 | 11.33 | 14.88 | 14.18 | 18.56 | 15.17 | 16.1 | 21.35 | 23.61 |
|  | PA | 8.39 | 15.63 | 10.86 | 16.16 | 11.41 | 14.44 | 13.96 | 15.91 | 14.22 | 15.98 |
|  | AP | 8.34 | 14.1 | 6.39 | 9.05 | 10.61 | 13.62 | 12.58 | 15.21 | 12.13 | 13.73 |
|  | TO | 8.14 | 13.87 | 9.66 | 13.41 | 11.6 | 13.79 | 15.4 | 16.9 | 15.65 | 16.98 |
| Northeast | MA | 4.01 | 10.14 | 7.89 | 12.25 | 10.44 | 13.54 | 11.24 | 13.13 | 12.03 | 14.22 |
|  | PI | 5.38 | 10.4 | 6.95 | 11.99 | 10.87 | 14.87 | 13.04 | 15.75 | 14.34 | 17.2 |
|  | CE | 10.71 | 15.76 | 14.86 | 19.68 | 18.65 | 22.73 | 22.16 | 25.2 | 21.84 | 24.15 |
|  | RN | 10.7 | 18.54 | 14.3 | 19.92 | 16.64 | 20.88 | 19.51 | 22.44 | 16.74 | 18.5 |
|  | PB | 7.98 | 14.35 | 14.12 | 18.99 | 15.05 | 18.38 | 15.75 | 18.21 | 17.67 | 20.18 |
|  | PE | 15.3 | 21.3 | 18.72 | 22.84 | 19.32 | 22.29 | 21.27 | 23.29 | 21.52 | 23.72 |
|  | AL | 8.99 | 13.7 | 10.45 | 14.77 | 12.23 | 15.93 | 14.46 | 17.85 | 15.65 | 18.99 |
|  | SE | 8.63 | 13.19 | 10.96 | 14.85 | 12.43 | 16.01 | 15.08 | 18.64 | 16.93 | 20.59 |
|  | BA | 11.16 | 15.68 | 13.42 | 17.72 | 15.18 | 19.51 | 17.27 | 20.9 | 17.83 | 20.52 |
| Midwest | MT | 11.24 | 14.54 | 14.51 | 17.74 | 17.09 | 19.09 | 18.22 | 19.49 | 19 | 19.02 |
|  | MS | 17.43 | 18.44 | 19.42 | 20.13 | 20.18 | 19.57 | 21.33 | 20.1 | 20.26 | 18.69 |
|  | GO | 13.39 | 17.92 | 14.64 | 18.98 | 17.78 | 21.32 | 20.72 | 25.63 | 20.11 | 24.05 |
|  | DF | 22.28 | 26.85 | 22.35 | 26.56 | 24.25 | 29.72 | 22.57 | 29.14 | 21.26 | 27.89 |
| Southeast | MG | 13.93 | 18.85 | 15.27 | 19.78 | 17.09 | 21.08 | 18.15 | 21.25 | 18.18 | 21.72 |
|  | ES | 16.52 | 17.98 | 17.4 | 17.93 | 19.22 | 20.18 | 21.56 | 22.75 | 22.5 | 24.26 |
|  | RJ | 18.28 | 21.19 | 19.07 | 21.89 | 20.79 | 23.65 | 22.82 | 25.95 | 23.37 | 27.05 |
|  | SP | 18.86 | 22.33 | 20.51 | 23.9 | 21.89 | 25.08 | 23.78 | 27.34 | 22.53 | 27.46 |
| South | PR | 19.44 | 23.5 | 19.85 | 23.18 | 20.79 | 23.69 | 21.47 | 23.77 | 21.91 | 24.86 |
|  | SC | 17.99 | 26.1 | 18.07 | 25.12 | 21.15 | 27.79 | 22.15 | 27.48 | 22.16 | 27.87 |
|  | RS | 25.12 | 26.01 | 24.28 | 24.15 | 24.36 | 22.68 | 24.81 | 21.81 | 24.75 | 21.63 |

**Table S1**. Age-Standardized Breast Cancer Mortality Rates Across Brazilian States: Uncorrected vs. Adjusted Data by Period, 2000–2023.

Notes: a) Age-standardized rates are expressed per 100,000 women, based on the World Health Organization standard population (2000-2025); b) Wilcoxon signed-rank test comparing uncorrected and adjusted rates across federative units (UFs) showed statistically significant differences for all periods (p < 0.001).
